# Supplementary material for: Myokine Musclin Is Critical for Exercise-Induced Cardiac Conditioning
Source: Int J Mol Sci. 2023 Mar 30;24(7):6525. doi: 10.3390/ijms24076525 (PMC10095193; doi:10.3390/ijms24076525)
Supplement: Supplementary file 1 [file ijms-24-06525-s001.zip › ijms-2230497-supplementary.pdf]

Supplemental materials

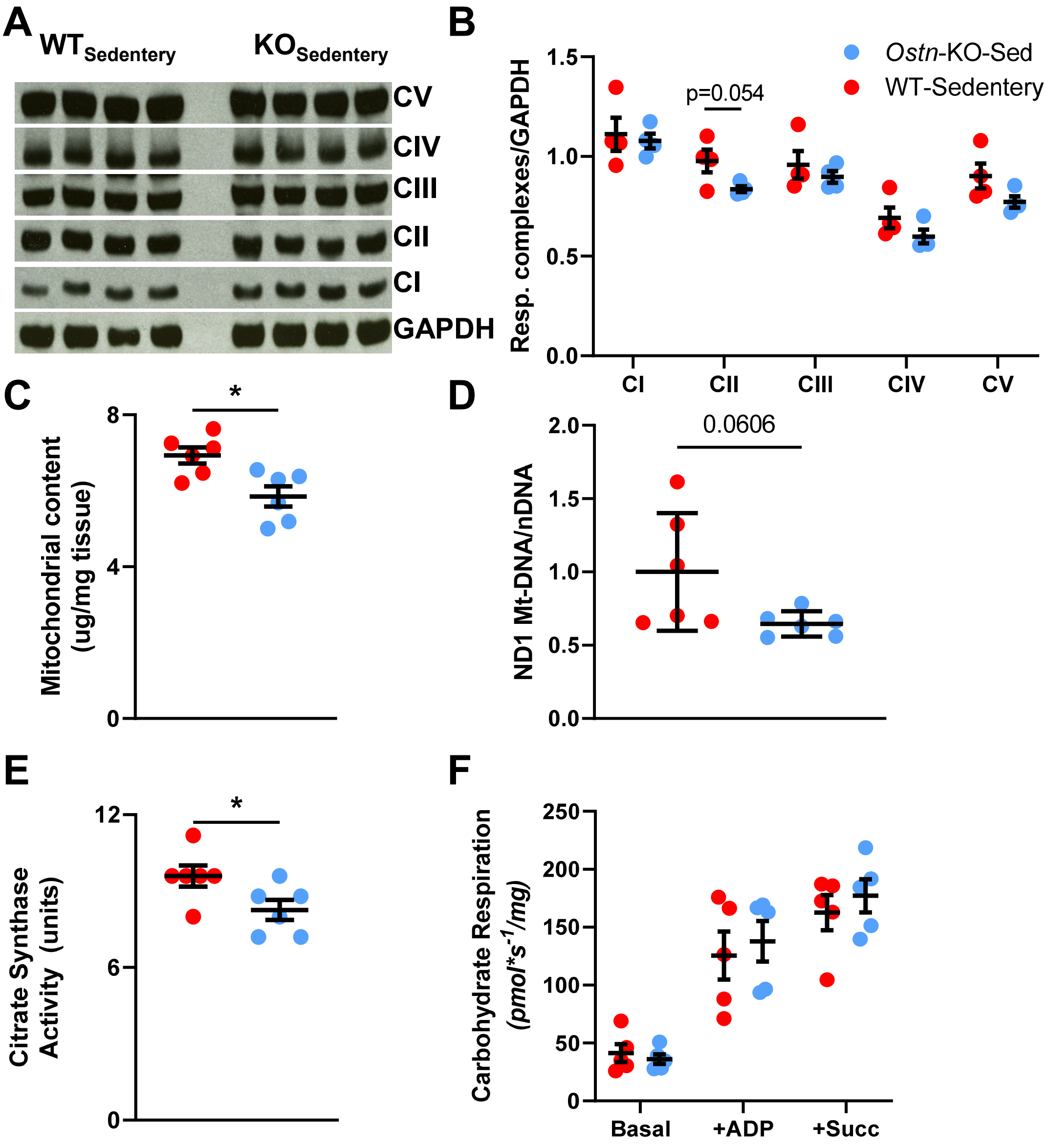

Figure S1. Mitochondrial biogenesis and respiratory capacity in hearts of *Ostn*-KO and WT control mice under "sedentary" (regular housing) conditions. (A) Representative western blots of respiratory chain enzymes and GAPDH and (B) summary statistics for respiratory complex expression normalized to GAPDH in hearts of sedentary WT and *Ostn*-KO mice (n=4 each): Complex I expression (1.08±0.08 *vs.*

1.11±0.17 AU, p=0.7), complex II expression (0.83±0.03 *vs*. 0.98±0.11 AU, p=0.054), complex III expression (0.89±0.06 *vs*. 0.96±0.14 AU, p=0.45), complex IV expression (0.6±0.07 *vs*. 0.69±0.1 AU, p=0.18), complex V expression (0.77±0.06 *vs*. 0.9±0.13 AU, p=0.11) (C) Summary statistics for mitochondrial content by tissue weight in hearts from sedentary WT and *Ostn*-KO mice (n=6 each, 6.67±0.60 *vs*. 5.85±0.66 µg/mg tissue, p=0.01). (D) Summary statistics for expression of ND-1 normalized to hexokinase 2 in hearts from sedentary WT and *Ostn*-KO mice (n=6 each, 1.00±0.40 *vs*. 0.65±0.09 AU, p=0.06). (E) Summary statistics for citrate synthase activity in mitochondrial extracts from hearts of sedentary WT and *Ostn*-KO mice (n=6 each, 9.59±1.01 *vs*. 8.26±0.97 AU, p=0.04). (F) Summary statistics for carbohydrate driven mitochondrial respiration in semi-permeabilized fibers normalized to tissue weight in hearts from sedentary WT and *Ostn*-KO mice (n=5 each): Under carbohydrate-supported conditions respiration in myofibers isolated from hearts of sedentary WT and Ostn-KO mice were comparable (basal: 41.39±17.18 *vs*. 36.21±9.41 pmol*s-1/mg, p=0.57, with ADP stimulation: 125.56±46.32 *vs*. 137.88±39.05 pmol*s-1/mg, p=0.66, and with addition of succinate: 162.63±33.89 *vs*. 177.17±31.87 pmol*s-1/mg, p=0.5). Data are means ± SD; *P<0.05.

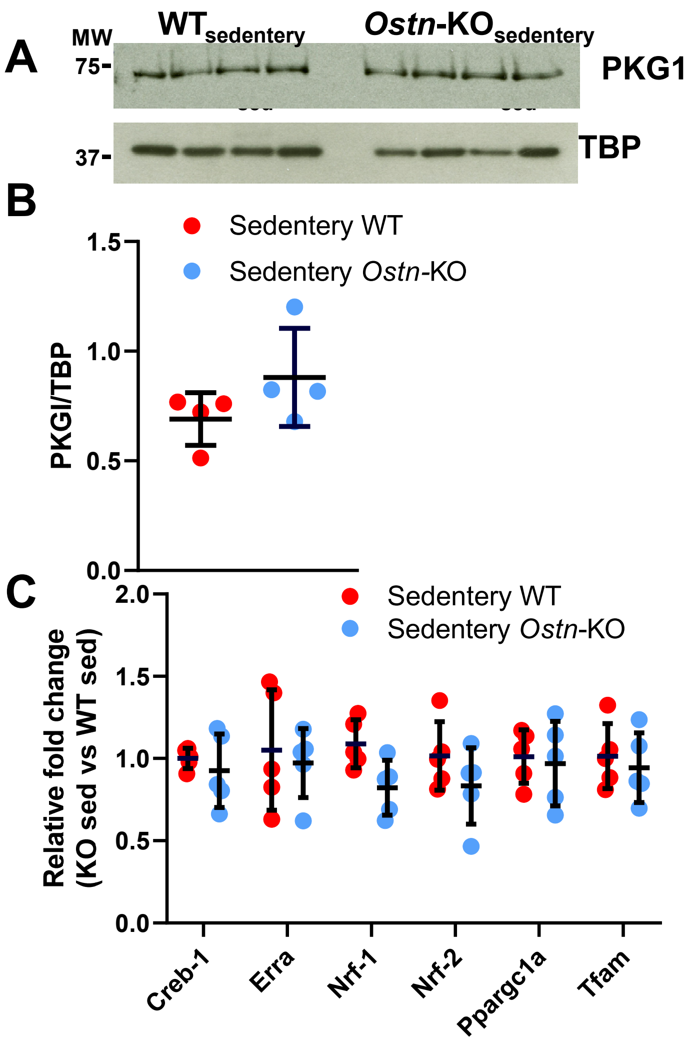

Figure S2. Nuclear protein kinase G1 expression and mRNA expression of mitochondrial biogenesis-related genes in *Ostn*-KO and WT mice under "sedentary" (regular housing) conditions. (A) Representative western blots of PKG1 and TATA binding protein in nuclear extracts from hearts of sedentary WT and *Ostn*-KO mice, (B) Summary statistics for PKG1 normalized TBP in nuclear extracts from hearts of sedentary WT and *Ostn*-KO mice (0.51±0.76 *vs*. 0.68±1.2, n=4, p=0.19). (C) Summary statistics for mRNA expression of *Creb1*, *Erra, Nrf1, Nrf2, Ppargc1a,* and *Tfam* in hearts from sedentary WT and *Ostn*-KO mice (n=6). Data are means ± SD.

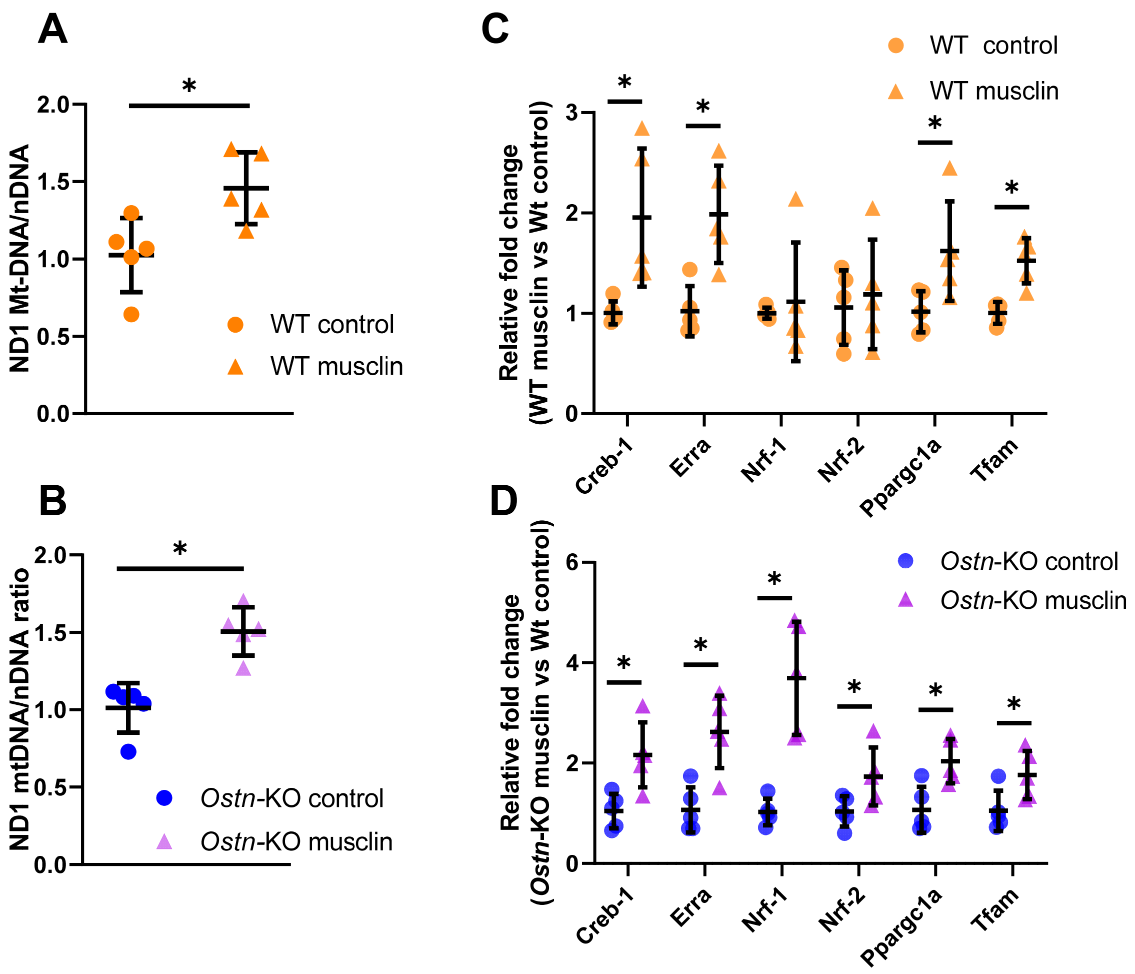

Figure S3. Synthetic musclin infusion promotes mitochondrial biogenesis. (A) Summary statistics for expression of ND-1 normalized to hexokinase 2 in hearts from sedentary WT mice infused with control peptide or synthetic musclin for 14 days (1.09±0.24 *vs*. 1.46±0.23, n=5 each, p=0.02). (B) Summary statistics for expression of ND-1 normalized to hexokinase 2 in hearts from sedentary *Ostn*-KO mice infused with control peptide or synthetic musclin for 28 days (1.01±0.16 *vs*. 1.51±0.16, n=5 each, p=0.001). (C) Summary statistics for mRNA expression of *Creb1*, *Erra, Nrf1, Nrf2, Ppargc1a,* and *Tfam* in hearts from sedentary WT mice infused with control peptide or synthetic musclin for 14 days (all n=5 each, *Creb1*: 1.01±0.11 *vs*. 1.95±0.69 fold change, p=0.02, *Erra*: 1.02±0.25 *vs*. 1.99±0.49 fold change, p=0.004, *Nrf1*: 1.0±0.06 *vs*. 1.12±0.59 fold

change, p=0.68, *Nrf2*: 1.06±0.3 7*vs*. 1.19±0.55 fold change, p=0.67, *Ppargc1a*: 1.02±0.2 *vs*. 1.62±0.49 fold change, p=0.04) , and *Tfam*: 1.01±0.11 *vs*. 1.52±0.22 fold change, p=0.002). (D) Summary statistics for mRNA expression of *Creb1*, *Erra, Nrf1, Nrf2, Ppargc1a,* and *Tfam* in hearts from sedentary *Ostn*-KO mice infused with control peptide or synthetic musclin for 28 days, n=5 each (*Creb1*: 1.05±0.34 *vs*. 2.16±0.65 fold change, p=0.009, *Erra*: 1.07±0.45 *vs*. 2.62±0.72 fold change, p=0.003, *Nrf1*: 1.03±0.27 *vs*. 3.695±1.12 fold change, p=0.001, *Nrf2*: 1.04±0.3 *vs*. 1.73±0.58 fold change, p=0.04, *Ppargc1a*: 1.07±0.46 *vs*. 2.04±0.44 fold change, p=0.009, and *Tfam*: 1.05±0.4 *vs*. 1.76±0.48 fold change, p=0.03). Data are means ± SD; *P<0.05,.
